# Changes in Spirometry Indices and Lung Cancer Mortality Risk Estimation in Concrete Workers Exposed io Crystalline Silica

**DOI:** 10.31557/APJCP.2020.21.9.2811

**Published:** 2020-09

**Authors:** Somayeh Rahimi Moghadam, Narges Khanjani, Mahmoud Mohamadyan, Mojtaba Emkani, Saeed Yari, Mohamad Naser Layegh Tizabi, Ali Ganjali

**Affiliations:** 1 *Department of Occupational Health Engineering, Neyshabur University of Medical Sciences, Neyshabur, Iran. *; 2 *Environmental Health Engineering Research Center, Kerman University of Medical Sciences, Kerman, Iran. *; 3 *Department of Occupational Health Engineering, School of Health, Mazandaran University of Medical Sciences, Sari, Iran.*; 4 *Department of Occupational Health Engineering, Faculty of Health, Social Determinants of Health Research Center, Gonabad University of Medical Sciences, Gonabad, Iran. *; 5 *Department of Occupational Health Engineering, Shahid Beheshti University of Medical Sciences, Tehran, Iran. *; 6 *Student Research Committee, Neyshabur University of Medical Sciences, Iran. *

**Keywords:** Concrete, pulmonary function, lung cancer, risk, silica dust

## Abstract

The health of workers in the concrete and cement industries can be at risk due to occupational exposure to silica dust. The purpose of this study was to evaluate the changes of pulmonary parameters and risk of mortality from lung cancer in concrete workers exposed to crystalline silica. This cross-sectional study was performed on 72 male workers exposed to silica at a concrete manufacturing plant in Neyshabur, Iran. Respiratory zone air sampling was performed using the standard NIOSH7602 method using individual sampling pumps and membrane filters. Then, the amount of silica in the samples was determined using the Fourier Transform Infrared technique. The risk of death from lung cancer was determined using Rice et al.’s model. Respiratory indices were measured using a spirometer. Data were analyzed by the SPSS 20 software. Occupational exposure to silica was 0.025 mg/m^3^ and mortality was estimated to be 7-94 per thousand. All spirometry indices significantly decreased during these 4 years of exposure to silica dust. The respiratory pattern of 22% of the exposed workers was obstructive and this prevalence was significantly higher than the control group. The results showed that although the average occupational exposure to silica in these concrete workers was below the recommended threshold of national and international organizations, their risk of death was significantly higher; and workers’ lung indices had significantly decreased over four years. Therefore, appropriate measures should be taken to reduce silica exposure among these workers.

## Introduction

Concrete is the product of the reaction between water and cement in special environmental conditions. The main material used in concrete producing factories is cement (Neville and Brooks, 1987). Increased construction activity in developing countries and increased demand for cement and concrete production, requires the employment of more workers in the cement and concrete factories; and therefore, more workers get exposed to dust (Mahmood et al., 2010). The volume of cement production is very high in Iran, and in 2009 it reached 600,000 tons (Poornajaf et al., 2010). 

A main occupational hazard in the cement and concrete industries is the production of fine particles during the industrial process (Omid et al.,2014). Workers in industrial sites are exposed to a mixture of dust produced from silica, cement and sand (Sumanaet al., 2016). 

Recent studies show that more than 2.3 million workers in 2016 in the United States (Occupational Safety and Health Administration, 2016), over 2 million in 2007 in Europe (Maciejewska, 2008) and over 23 million in 2009 in China had occupational exposure to crystalline silica (Chen et al., 2012). The International Agency for Research on Cancer classified Crystalline silica as carcinogenic to humans (Classified at group 1A) in October 1996 (OSHA, 2016). Workers in different industries such as casting, stone grinding, construction, tile making, glazing, sandblasting and statue making are exposed to crystalline silica dust and this can seriously endanger their health (Mohammadi et al., 2017).

Numerous studies have shown that chronic exposure to over permissible levels of dust causes lung diseases such as pulmonary fibrosis, lung cancer and decreased respiratory capacity (Hnizdo and Vallyathan, 2003). The rate of pulmonary functions decline in workers exposed to silica dust is high and they are likely to develop occupational asthma, chronic bronchitis, and silicosis (Chen et al., 2006). Sakar et al., (2005) showed that tile workers are at risk of developing silicosis and this risk increased with increase in age and duration of contact.

Aminian et al., (2012) conducted a retrospective cohort study in 2011 on 182 people including 95 workers exposed to cement, silica and lime dust in the cargo department as cases and 93 administrative personnel as controls. Their study showed that the prevalence of respiratory symptoms such wheezing, dyspnea and coughing in the exposed group was significantly higher than the control group. Also, in workers who had a higher exposure to cement dust, a significant drop in spirometry indices including (Forced Vital Capacity) FVC, (Forced Expiratory Volume in the first second) FEV1, FEV1/FVC, (Forced Expiratory flow) FEF 25-75 and (Peak Expiratory Flow) PEF were seen, and there was a higher prevalence of obstructive pulmonary disease in the exposed group. 

Seifaghaie (2000) did not see a significant difference in the spirometry indices of 263 workers exposed to Portland cement in Jajrood, Iran compared to 99 controls who were office employees. Respiratory symptoms were not seen in some other studies either (Mehrparvar et al., 2011; Meo, et al., 2013; Mohammadi and Tajdinan, 2010; Parsi, 2011)

Concrete workers are exposed to dangerous material such as cement and silica dust. The health of workers is ethically and economically important because, in societies such as Iran, production and economic gain are related to worker’s health. Therefore, in order to have a vivid economy, it is necessary to pay attention to the worker’s health. This study was conducted in order to inform workers and officials and to persuade them to pay more attention to worker’s health and improve their working conditions. 

In recent years, risk assessment has become one of the most important topics in occupational disease control. Nowadays, not only the toxicity or side effects of exposure to chemicals are important, but also the mortality and morbidity associated with them. The aim of this study was to evaluate changes in spirometry indices and the risk of mortality from lung cancer in concrete workers exposed to crystalline silica.

## Materials and Methods

*The population under study*

This was a cross-sectional study conducted in one concrete making factory in Neyshabour, Iran in 2015. All workers exposed to dust were enrolled. There were 100 workers working in the concrete making factory. After applying the inclusion and exclusion criteria, 72 workers were enrolled in the study. Air sampling and spirometry tests were done for all 72 workers.

*Measurement of silica dust*

Occupational exposure to crystalline silica dust was measured based on the NIOSH7602 method (National Institute of Occupational Safety and Health, 2003) by using a personal 10 mm nylon cyclone sampling pump, and a 25 mm sampling membrane filter with a 0.8 μm pore size, made by SKC, UK. Initially, the sampling pump was calibrated with a soap bubble burette according to the recommended flow rate of 1.7 L/min. The filters were inserted in the desiccator for 24 hours before and after sampling. Then they were weighed with a precision of 0.00001 grams before and after sampling. Sampling was performed at a rate of 1.7 L/min during the shift. In order to eliminate the bias during sampling, for every 5 samples taken, one control was considered.

After sampling, the filters containing the samples and controls were transferred to the laboratory and the samples were analyzed using infrared spectrophotometry (IR) which has high sensitivity and reliability to measure crystalline silica. 

Quartz was purchased from Merck Germany and was crushed in the grinder into particles in the size range of 10 to 50 microns. Because particles under 20 microns were required, the powder was filtered and particles under 20 microns were separated. The purity of the sample was quantitatively and qualitatively analyzed by the X-ray diffraction (XRD) (model PW1800) and the X-ray fluorescence (XRF) (model PW1480) spectrometer manufactured by PHILIPS Netherlands (National Institute of Occupational Safety and Health, 2003).

Then, the filters containing samples were placed in a crucible, and 200 mg of potassium bromide (made by Merck Germany) was added to the filters. Then they were heated in a muffle furnace (made by Lenton, England) for 2 hours at 600°C. After cooling, the sample was homogenized in a mortar and the tablet was prepared in metal frame 13, using a press machine at a pressure of 20 MPa.

Standard pure inhalable quartz dust samples are needed in concentrations lower than and above permissible values, in order to draw the calibration curves. Standard samples were prepared with 1, 1.5, 3.5 and 4 μgr of inhaled quartz with 80% purity. Standard specimens were prepared at the highest absorbance peak (wavelength 810 m-1) in the reading machine (FTIR technique, WQF-A510FT) and the standard curve of quartz was prepared. The standard curve was used for quantitation of inhalable quartz dust in the samples obtained from the worker’s respiratory zone.

Finally, the concentration of free silica in workers’ respiratory zone was calculated by Equation 1 (NIOSH, 2003).

*Equation 1*

C=(A-B)/(M×V)

C is the concentration of crystalline silica in milligrams per cubic meter, A is the absorbance in the original sample, B is the absorbance in the control sample, M is the slope of the calibration curve, and V is the corrected volume of sampled air (L).

*Risk Assessment*

Rice and co-workers have presented a model to assess the risk of lung cancer in workers exposed to crystalline silica. They predicted a risk model for lung cancer mortality, assuming exposure to the standard OSHA level for crystalline silica, 45 years of continuous work and a 10-year time lag.

The likelihood of lung cancer mortality was calculated using the linear regression model from Rice et al., ( 2001).

*Equation 2*

*Excess Death because of Lung Cancer*

= *0.77+373.69×Geometric Mean of Exposure*

*Evaluation of Pulmonary Indices*

In addition to the spirometry’s done on workers in 2015, three other spirometry’s had been done in the previous three years (2012, 2013, and 2014) and this information was extracted from the medical records. Eventually, changes in the respiratory function indices of workers exposed to dust during these four years (2012-2015) were evaluated. Spirometry indices were measured in all the years by a reputable occupational medicine company with the same devices; and the type or model of the spirometer did not change.

The criteria for entering the study were the workers’ consent and at least 4 years of work experience in this industry. The exclusion criteria were having chronic pulmonary diseases, asthma, a history of chronic respiratory infection or any thoracic surgery and a history of cardiac disease, before employment in the cement factory. Workers who worked in other high-risk occupations before this job or had a second job which could have affected their spirometry’s after these years were excluded from the study. 

Information such as age, height, weight, BMI, work experience and smoking status was asked from the workers and recorded. The spirometry test included FVC, FEV1, FEV1/FVC, FEF 25-75 and PEF which were performed according to the guidelines of the American Thoracic Association (Rahimi Moghaddam & Khanjani, 2014) and with a portable calibrated spirometer (COMPACT model, made by The Vitalgraph Company) in similar situations for all workers, and was done by a trained technician and under the supervision of an occupational physician. 

In order to do the spirometry, first information such as name, age, gender, height, weight and race was recorded, then a nose clip was applied and participants were asked to put the mouthpiece in their mouth, and after 2-3 ordinary inhaling and exhaling’s, they were asked to do a deep inhaling and then do a quick and strong exhale which lasted about 6 seconds. This test was done for each person at least 3 to 8 times. The percent predicted for each of the functional respiratory parameters according to age, height, gender and race were calculated by the spirometer. Participants were asked to refrain from bathing at least 2 hours before the respiratory test and not to use drugs effective on respiratory function 24 hours before the spirometry test. Participants were familiarized with the spirometry device and the maneuvers beforehand. 

Eventually, the spirometry and demographic data were entered into SPSS 20. The Kolmogorov-Smirnov test was used to check data normality. Data were summarized by descriptive statistics and analyzed by repeated measures tests. P-values of less than 0.05 were considered significant.

## Results

The study population consisted of 72 male concrete workers exposed to cement and silica dust with an average age of 38.91 ± 5.96 and a work experience of 6.58 ± 1.74 years. 25% of them were smokers and none of the workers used respiratory protection gear. Other demographic characteristics are presented in Table 1.

*Silica monitoring and lung cancer mortality risk estimation*

The amount of exposure to crystalline silica dust among concrete workers was between 0.025 ± 0.008 mg/m^3^. The highest exposure to silica was in the autoclave unit, wing tube and cutter line and was 0.08, 0.07 and 0.06 mg/m^3^, respectively. The lung cancer mortality risk was 7 to 94 per thousand in these silica-exposed workers, according to the model of Rice et al., (2001). Table 2 summarizes exposure levels to crystalline silica among different occupational groups and their risk of lung cancer mortality.

*Spirometry Results*

The respiratory indices were also compared across the 4 years under study, among the workers. The mean of respiratory indices from 2012 to 2015 has been shown in Table 3. The results showed that all indices decreased significantly.

According to the inclusion and exclusion criteria all those included in the study, were healthy individuals free of diseases and medications affecting lung parameters, at the beginning. Their disease patterns were examined at the end of this study which was 2015. The frequency of the obstructive pattern in the exposure group was 16 participants (22%) and the rest had a normal pattern.

The results showed that exposure to silica dust was 0.025 mg/m^3^ in these concrete workers. The occupational exposure threshold to crystalline silica is 0.025 mg/m^3^ as recommended by the American Conference of Governmental Industrial Hygienists (American Conference of Governmental Industrial Hygienists, 2019) and the Iranian Occupational Health Committee (Ministry of Health and Medical Education, 2017). In general, the average exposure in this study was close to the recommended national and international standard. The exposure range was 0.008 to 0.08 mg/m^3^, and the workers of the autoclave, wing tube and cutting line had values above the standard threshold. In a study aimed at assessing the risk of exposure to silica in construction workers in eastern Tehran, Iran, Azari et al., (2009) showed that their exposure was 0.13 mg/m^3^ and above the standard threshold. In Parsaseresht et al., (2016)’s study about assessing occupational exposure to crystalline silica in sand washing workers, the average exposure to crystalline silica was 0.21 mg/m3 and above the occupational exposure threshold. The mean exposure to respiratory crystalline silica in Zarei et al. study on 55 foundry workers was 0.246 ± 0.04 mg/m^3^, and all workers’ exposure was above the international standard limit (Zarei et al., 2017).

**Table 1:** The Demographic Characteristics of the Participating Workers

Variable	Mean±SD
Age (year)	38.91±5.96
Height (cm)	175.8±7.49
Weight (kg)	76.72±12.76
Body Mass Index	24.97±4.98
Work experience (year)	6.58±1.74
	Frequency (Percent)
Smoking	
Yes	18 (25)
No	54 (75)
Use of protective device	
Yes	10 (14.28)
No	60 (85.72)

**Table 2:** Occupational Exposure and Lung Cancer Mortality Risk among Workers Exposed to Silica in Different Occupational Groups

Occupational group	Mean exposure±SD (mg/m^3^)	Estimation of lung cancer mortality risk (per thousand workers)
Wing Tube	0.07±0.01	26.92
Mixer	0.024±0.009	9.73
Autoclave	0.08±0.02	30.66
Packing	0.009±0.002	4.13
Quality Control (QC)	0.008±0.002	3.75
Cutting Line	0.06±0.03	23.19
Laboratory	0.008±0.002	3.75
Repair Section	0.009±0.003	4.13
Total	0.025±0.008	10.11

**Table 3:** The Mean of Respiratory Indices in the Exposed Group from 2012 until 2015

Indices	2012	2013	2014	2015	*P-value
Mean ±SD					
FVC (L)	4.12±1.54	4.04±0.43	3.97±0.61	3.93±0.89	0.005
FEV1 (L)	3.79±0.51	3.65±0.21	3.57±0.33	3.55±0.50	0.001
FEV1/FVC%	91.9±6.27	90.34±6.89	89.92±5.58	90.3±6.43	0.02
PEF (L/S)	9.8±1.09	9.5±1.88	9.4±2.01	9.1±1.12	0.004
FEF25-75 (L/S)	3.88±0.76	3.75±0.67	3.62±1.11	3.47±1.15	0.003

*Repeated Measures test

## Discussion

These different results can be related to the nature of different jobs and the amount of dust produced in each occupation, as well as the technology used in these industries (e.g. using advanced and fully automatic devices with the least human interference or not) and the hours and duration of exposure. The amount of silica dust production depends on the industries. The industry studied in this article was an advanced industry, with most of the work done by fully automatic machines and the workers did minimum physical work.

According to Iran’s Labor Law, Article 91, employers are obliged to provide workers with the necessary equipment and facilities to ensure the safety and health of workers in the workplace. Workers are also obliged to use and take care of personal protective equipment in their working place. 

But unfortunately, in most industries, low quality equipment is provided solely for the purpose of obeying these laws, and some workers do not regularly use personal protective equipment, because the equipment has low quality, or is heavy or cumbersome.

A study was conducted about the attitude, safety behavior and personal protective equipment use in employees of Isfahan, Iran, metro stations and results showed that the most commonly used safety and personal protective equipment were safety shoes and gloves. But, safety helmets, anti-dust masks, and proper work clothes were rarely used (Shamsi et al., 2013).

Another result of the present study was a significant decrease in all workers’ respiratory indices. Concrete workers are exposed to various inhalable dusts that enter deep into the lungs and cause inflammation in the mucosal membrane of the lungs and abnormal respiratory function tests (Sumana et al., 2016). Meo et al., (2013)’s study reported that workers who were exposed to cement dust for 5-10 years showed a significant decrease in FVC, FEV1 and MVV indices in comparison to the control group. But in the FEV1/FVC, PEF, FEF25-75% indices there were no significant differences between the two groups. 

A cross-sectional study conducted by Gholami et al., (2007) on 156 kaolin and gold miners and stone cutting workers and 48 controls; showed that the mean of lung function parameters including FVC and FEV1 was lower among the workers than the controls (P<0.05), but there was no significant difference between the two groups in peak expiratory flow and forced expiratory flow 25-75% (P>0.05). 

The result of these studies confirms the results of our study and show that continuous exposure to dust will cause a decrease in the spirometry indices. Several studies have confirmed that occupational exposure to cement dust is an important risk factor for chronic pulmonary diseases (Poornajaf et al., 2010). Mahmood et al., (2010) also found a significant decrease in the PEF among the workers of a cement factory who were exposed to dust, but their other spirometry indices had not decreased significantly. Also, the results of Poornajaf et al., (2010)’s study in Iran showed that workers exposed to cement dust in comparison to controls had a significant decrease in FVC, FEV1, FEV1/FVC indices. 

However, in some studies no significant difference has been reported in regard to pulmonary function tests including FVC, FEV1, FEV1/FVC or respiratory symptoms in workers exposed to cement (case group) and other workers (controls) (Aminian et al., 2012; Baum et al., 1998; Mohammadi and Tajdinan, 2010). This difference is probably related to using or not using personal protective devices, the amount of exposure to dust, length of exposure, risk management methods, working shift, movement of workers, surface of the working place and availability of natural or mechanical ventilation equipment. 

The results of the present study showed that 22 percent of workers had an obstructive pattern of lung dysfunction. One of the abnormal spirometry patterns is the obstructive pattern. The most prominent characteristic of the obstructive pattern is decrease in the speed of exhalation (FEV1/FVC). In obstructive pulmonary diseases, FEV1 and the percent of FEV1/FVC decrease (Miller et al., 2005). Silica has been known as a risk factor for obstructive pulmonary diseases (Hnizdo and Vallyathan, 2003; Rushton, 2007). Aminian et al., (2012)’s study showed that the prevalence of obstructive pulmonary disease was significantly higher in workers exposed to cement and silica than unexposed people (28% vs 10%). In Mahmood et al., (2020)’s study, in workers with direct exposure to cement dust, 3.1%; and in workers with indirect exposure, 2.4% showed obstructive pulmonary disease patterns. 

In this study, the average risk of lung cancer mortality was 10.11 per 1,000 workers with a range of 3.75 to 30.66. In a study done by Normohammadi et al., (2016) lung cancer mortality rates in silica exposed workers were estimated to be 32-60 per thousand, Azeri et al., (2017)(2009) estimated lung cancer mortality between 21 to 49 per 1,000. Chen et al., (2006) investigated the causes of deaths among 7,000 Chinese miners exposed to silica and its mixed particles and found that death from lung cancer was the third leading cause of death among workers and accounted for 7% of all deaths. In another cohort study, Liu et al., (2013) assessed the risk of deaths from exposure to silica among 34,000 workers over 44 years and reported 542 deaths due to lung cancer during this period.

Omidianidost et al., (2016) estimated the mortality rate of silicosis in small castings workshops to be 1-13.7 per 1,000 people; and mortality from lung cancer was also reported to be 4-16 per 1,000 people, assuming 45-years of exposure. Also, exposure to crystalline silica in 50% of workers in the study sites was above the permissible exposure level. In the study conducted by Zarei et al., (2017) on casting workers, the risk of mortality from silicosis was estimated based on the Mannetje model and was in the range of 6 to 63 per 1,000 persons. All workers were above the acceptable risk level, which is 1 in every 1,000 people according to the OSHA criteria; and increased mortality from lung cancer due to crystalline silica, based on the Rice model, assuming 45 years of continuous exposure was estimated to be 65 per 1,000 people, ranging from 19 to 897.

The results of the present study and the above studies indicate the high possibility of lung cancer mortality among people exposed to silica dust and indicate the importance of attention to silica exposure control and regular monitoring to prevent cancer.

One of the limitations of this study was the low sample size and lack of a control group. It is recommended that studies with larger sample sizes and with control groups be conducted over longer years.

In conclusion, the results of this study indicate, that although the average exposure of concrete workers to crystalline silica dust was within the recommended range of national and international standards; but some workers were exposed to levels above the recommended occupational standards and are expected to have a high mortality rate from lung cancer. These workers’ respiratory indices also decreased significantly over four years.

This fact requires the attention of managers to protect the health of workers and control dust in workplaces by assembling proper ventilation devices in polluted saloons, and also providing proper high-quality personal protective devices for workers. Additionally, educating the workers about proper use of the devices; and supervision on their correct and constant use of protective equipment is necessary. Engineering and health control measures can improve the industrial process and reduce exposure and prevent silicosis, lung cancer and other potential complications, and also reduce the cost of medical care.
